# How to decide? Different methods of calculating gene expression from short oligonucleotide array data will give different results

**DOI:** 10.1186/1471-2105-7-137

**Published:** 2006-03-15

**Authors:** Frank F Millenaar, John Okyere, Sean T May, Martijn van Zanten, Laurentius ACJ Voesenek, Anton JM Peeters

**Affiliations:** 1 Plant Ecophysiology, Institute of Environmental Biology, Faculty of Science, Utrecht University, Sorbonnelaan 16, 3584 CA Utrecht, The Netherlands; 2 Nottingham Arabidopsis Stock Centre (NASC), Plant Science Division, University of Nottingham, Sutton Bonington Campus, Loughborough LE12 5RD, UK

## Abstract

**Background:**

Short oligonucleotide arrays for transcript profiling have been available for several years. Generally, raw data from these arrays are analysed with the aid of the Microarray Analysis Suite or GeneChip Operating Software (MAS or GCOS) from Affymetrix. Recently, more methods to analyse the raw data have become available. Ideally all these methods should come up with more or less the same results. We set out to evaluate the different methods and include work on our own data set, in order to test which method gives the most reliable results.

**Results:**

Calculating gene expression with 6 different algorithms (MAS5, dChip PMMM, dChip PM, RMA, GC-RMA and PDNN) using the same (Arabidopsis) data, results in different calculated gene expression levels. Consequently, depending on the method used, different genes will be identified as differentially regulated. Surprisingly, there was only 27 to 36% overlap between the different methods. Furthermore, 47.5% of the genes/probe sets showed good correlation between the mismatch and perfect match intensities.

**Conclusion:**

After comparing six algorithms, RMA gave the most reproducible results and showed the highest correlation coefficients with Real Time RT-PCR data on genes identified as differentially expressed by all methods. However, we were not able to verify, by Real Time RT-PCR, the microarray results for most genes that were solely calculated by RMA. Furthermore, we conclude that subtraction of the mismatch intensity from the perfect match intensity results most likely in a significant underestimation for at least 47.5% of the expression values. Not one algorithm produced significant expression values for genes present in quantities below 1 pmol. If the only purpose of the microarray experiment is to find new candidate genes, and too many genes are found, then mutual exclusion of the genes predicted by contrasting methods can be used to narrow down the list of new candidate genes by 64 to 73%.

## Background

Affymetrix GeneChip arrays have become a widely used microarray platform. An advantage of such a broadly accepted platform is that it greatly helps to compare RNA expression patterns between experiments from different researchers. RNA expression patterns are a starting point for high-level analysis such as clustering, self-organising maps or metabolic pathway analysis. The importance of "low level analysis", that is, calculating the expression levels and the normalization procedure as explained below, is easily underestimated.

Calculating gene expression is more complex with Affymetrix arrays compared to spotted arrays, as the latest generation Affymetrix arrays have 11 probe pairs available to interrogate each gene, whereas spotted arrays in general have only one cDNA or oligo probe per gene. Ideally, the signal intensity from all of these 11 probes should be equal because they all interrogate the same gene. In reality, however, there are enormous differences between individual probes in a probe set. The pattern of the probe signals in a probe set is called a "probe response pattern" [1]. The probe response pattern of one gene is roughly the same from all RNA samples isolated from a given tissue, irrespective of the treatment [1]. The Affymetrix system uses Microarray Suite (MAS) or its successor GeneChip Operating Software (GCOS) for operation of the microarray scanner and also for calculation of the expression values. The expression values are calculated according to the so-called "One-step Tukey's biweight algorithm", which is based on a weighed median [2]. Recently, more methods have become available for calculating gene expression values, such as "Model-Based Expression Indexes" (MBEI) from Li and Wong [1,3] that are implemented in the freeware program dChip. Also available is the "Robust Multi-array Average" (RMA) software from Irizarry *et al*. [4,5]. In contrast to MAS 5.0 or GCOS, in which information from only one microarray is used, model-based algorithms incorporate information from multiple microarrays to calculate the expression of a gene. The probe response pattern is fitted over multiple arrays with a multiplicative model in dChip and an additive model in RMA software. These algorithms use the fitted models to detect abnormally behaving probes, which are subsequently excluded for calculating gene expression. Therefore, gene expression from these model-based algorithms can be expected to provide more reproducible results. Both dChip and RMA use a stochastic model to estimate gene expression. A potentially better way of estimating gene expression would be the use of a physical model, that is, a model that explains the probe response pattern by physical laws. A few attempts have been made to make such physical models [6] such as "Positional-Dependent-Nearest-Neighbour model" (PDNN, [7]), or a combination of the two model types GeneChip and RMA (GC-RMA, [8]).

A specific feature of Affymetrix microarrays are the so-called MisMatch (MM) probes. Each of the 11 probe pairs in a probe set has a Perfect Match (PM) and a MisMatch probe, with each probe having 25 bases. The PM probes are designed to bind perfectly to the gene of interest and the MM probes have a contrasting base at position 13 with the intention to measure non-specific binding [9]. The PM and MM probe of one probe pair are located physically next to each other on the microarray. Both MAS5 and dChip in the PMMM mode use this information to calculate gene expression. However, in the literature there is a debate over the usefulness of MM probes, which has resulted in new algorithms that use only the PM signals to calculate gene expression. These algorithms are implemented in dChip PM mode [1,3], in the "Robust Multi-array Average" (RMA) software [4,5], GC-RMA [8] and in PDNN from Zhang *et al*. [7].

When dealing with experiments involving multiple arrays, there is a need for data normalization, after calculating gene expression. The overall signal intensity can vary between arrays and if this variation does not have a biological origin, it should be removed. Non-biological variation can arise due to differences in sample preparation, production and processing of arrays. There are different algorithms available to normalize microarray data. The MAS 5.0 or GCOS software currently assumes that intensity differences between two or more arrays are linearly related and have a zero y-intercept. This allows for a very simple and robust normalization factor. However, the drawback is that this method cannot adjust for non-linear relations. Schadt *et al*. [10] found 10 to 50% differences in the slope between the low and high expressed genes in many arrays. In those cases an alternative normalization method is needed. For example, dChip uses a non-linear smoothing method [3]. In MAS5 and dChip one array is chosen as the base line array, to which the other arrays are normalized. RMA does not use a base line array, but "quantile normalization", based on the complete data set [8]. Furthermore, it is preferred that genes changing in expression should not be included in the normalization procedure. Therefore, some algorithms (like dChip) use invariant sets; these comprise a set of probes that most likely have the same expression between arrays. An invariant set is determined by ranking probe intensities such that when a probe has approximately the same ranking number in two arrays, then this probe is likely to have the same expression in both arrays [1,3].

Thus different algorithms calculate gene expression and normalize the data in completely different ways. We hypothesize that the signal of gene expression, and therefore the identity of genes that are significantly up/down regulated, depends on the algorithm used. To test this hypothesis a microarray experiment was performed with *Arabidopsis thaliana *accession Columbia-0 and various calculation methods were compared. Plants were exposed for 3 hours to elevated ethylene levels (5 μl l^-1^) or were transferred to low-light levels for 3 hours (from 200 to 15 μmol m^-2 ^s^-1^). Both conditions induce an identical phenotype; the upward bending of the leaves by 20 degrees (hyponastic growth; [12]). The treated and control petioles were harvested at the same time with three biological replicates. The dataset is publicly available [13]. These data were analysed with six different methods: MAS5 (or GCOS), dChip PMMM, dChip PM-only, RMA, GC-RMA and PDNN. Some other studies have compared different calculation methods. Barash *et al*. [14] compared only three algorithms with a very limited amount of microarrays. Furthermore, Choe *et al*. [15] compared many options in the Bioconductor's Affy package, including the calculation of the expression values, however, again only three algorithms were compared. Seo *et al*. [16] compared six methods, four of which are also used in this paper.

Different algorithms are optimised for different experimental data sets and hence to different signal to noise ratios. Thus, the best method can depend on the specific experiment, so there might not be a perfect method suitable for all conditions [16]. It is therefore, important to test for each experiment which method is the most appropriate.

Since different calculated expression patterns were expected between algorithms, various criteria were designed and tested to compare the different methods, and to select the best method. 1: We compared the performance of the six methods on the spike-in data set of Affymetrix [17]. 2: Theoretically, we expected that model-based algorithms would produce more reproducible results because aberrant probes are efficiently removed. Therefore, we compared the distribution of the coefficient of variation for three replicated microarrays between the algorithms. 3: On the basis of data from the literature or previous experiments we expected that specific genes are affected by a treatment or mutation (biological relevance). 4: Furthermore, the problems with the use of MM signals as discussed in the literature are mainly statistical. Based on the following arguments we would like to discuss the use of MM values. i: First, we expected that cRNAs smaller than 13 base pairs bind perfectly to both probes. Calculation of the melting temperature is performed to estimate the likelihood of those events. ii: Secondly, we expected no correlation between the signals from the MM probes and the PM probes because the non-specific binding should have a random influence on the MM probes. To test this, we calculated correlation coefficients between PM and MM signals of 200 randomly chosen probe sets. 5: Microarray-obtained gene expression of a subset of the common genes was compared with Real-Time RT-PCR. Furthermore, we compared the expression from genes which are exclusively predicted with the best method.

The proposed criteria for biological relevance and reproducibility are easily applied by any microarray user on their own dataset. On the basis of all of the criteria described above a choice for the most suitable algorithm for each dataset is rationalized.

## Results and discussion

### Differences between six array algorithms

We hypothesized that the calculated level of gene expression is dependent on the algorithm used because these are based on completely different ways of calculating gene expression and normalization. As a result, a different set of genes may be identified as differentially expressed between a treatment and control.

**Figure 1:**
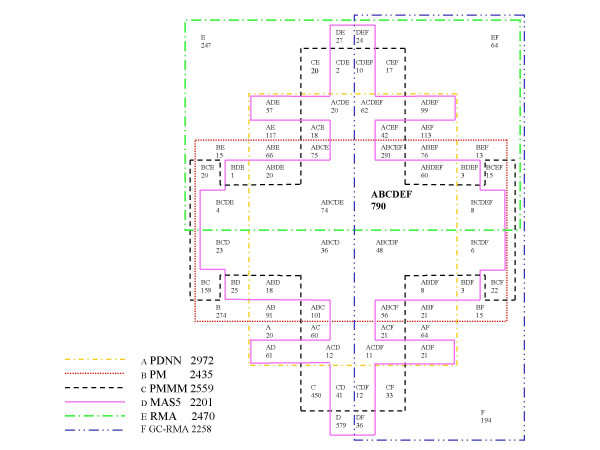
**Venn diagram**. Venn diagram of genes significant (Ttest p < 0.05) up or down regulated after three hours of ethylene exposure, depending of the method used to calculate gene expression. This diagram shows exactly the differences and similarities between all the methods. PDNN, MAS 5.0 (MAS, or GCOS), dChip PMMM (PMMM), dChip PM only (PM), RMA and GC-RMA were used. Only 790 genes were in common for all four algorithms. Comparable results were obtained from the low-light treatment. Areas with one letter shows genes which are unique for one method, areas with two letters shows genes which are only in common between these two methods, and so on.

After calculating gene expression with MAS5 (or GCOS), dChip PMMM, dChip PM, RMA, GC-RMA and PDNN, a T-test was performed to analyse differences in expression between plants treated with ethylene and air control. Between 2201 and 3262 genes were significantly differentially expressed (Table 1). These results showed that there were differences between the six algorithms in the number of genes differentially expressed within one treatment. However, the differences between the algorithms were relatively small. More strikingly, only 27% to 36% (790 genes) of the differentially expressed genes between control and ethylene treated plants were identical in the six methods (Fig. 1). Similar results were obtained when control and low-light conditions were compared (data not shown). This means that the total number of genes expressed differentially as calculated with these six algorithms was within the same order of magnitude, but the six algorithms identified totally different sets of genes!

When instead of a T-test with p < 0.05 more stringent conditions were used (p < 0.01; < 0.001; < 0.0001), less genes were found to be significantly regulated (2483; 798; 137; 21, respectively) and also the percentage genes significantly regulated in all algorithms decreased (32%; 16%; 2%; 0%). Moreover, if genes were selected that showed more than twofold up or down regulation, different numbers were obtained. However, the huge differences between the algorithms remained (data not shown). The set of genes that was detected by all methods had on average a lower p value (0.013) than the genes identified solely by one method (p = 0.028). On average the percentage genes that overlapped was 61% over all possible double combinations but dropped to 48 (all triple combinations), 40 (4), 35 (5) and finally to 32% (6). Our results showed a poor overlap between algorithms. This is not in full agreement with Barash *et al*. [12] and Choe *et al*. [14] who showed 60 to 70% overlap between algorithms. However, their results were based on a small number of microarrays or on a special (only) spike-in experiment. To our knowledge this is the first time that such large differences between algorithms are reported.

From the Venn diagram (Fig. 1) it is clear that MAS5 has the highest absolute number of uniquely identified differentially expressed genes compared to the other methods. When expressed relatively, MAS5 had the highest percentage of unique genes (26%), followed by PMMM (18%), PDNN (12%), PM (12%), RMA (10%) and GCRMA (9%). Furthermore, there are six possible combinations in which five methods might identify the same genes (Fig. 1; abcde, abcdf, abcef, abdef, acdef, bcdef), and the only combination in which MAS5 was not included (abcef) contains dramatically more genes (291) than the other five combinations in which MAS5 was included. These data show that MAS5 gives more unique results compared to the other five methods (Fig. 1).

**Figure 2:**
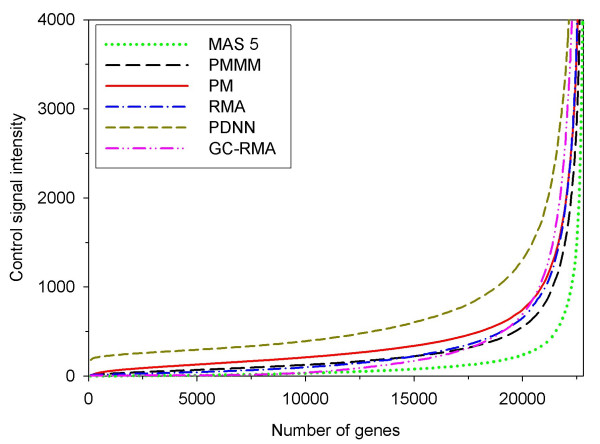
**Gene signal intensity**. Gene signal intensity from control plants of all genes (22747) calculated with six methods, MAS 5.0 (MAS), dChip PMMM (PMMM), dChip PM only (PM), RMA, GC-RMA and PDNN. The signal intensity is sorted from low to high. Similar results where observed with expression data from treated plants.

**Figure 3:**
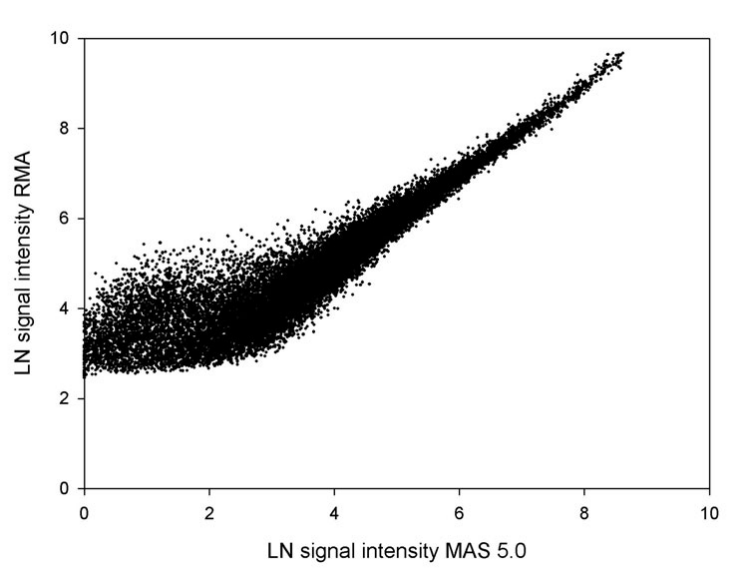
**Relation between signal intensity from MAS 5.0 and RMA**. Relation between the signal intensity calculated with MAS 5.0 and RMA software of all probe sets. In general there is a good correlation (r^2 ^= 0.9913), see also table 1. However, variation increased closer to the unity. For example a signal of 4 in MAS 5.0 results in a signal between 4 to 5.5 in RMA on a ln scale.

The differences observed between the six methods might have resulted from differences in calculated gene expression values between algorithms. MAS5 gave the lowest signals and PDNN the highest (Fig. 2). Overall there was a good correlation (r^2 ^= 0.9325 – 0.9913) between the signal intensities calculated with the different methods (Table 2, Fig. 3). However, despite this good overall correlation, large local variation remained (Fig. 3). For example, a gene with an expression of 100 (4.6 ln) in MAS5 may have an expression value that ranges between 120 (4.8 ln) and 500 (6.2 ln) in RMA. This variation between methods can explain partly the differences found between the algorithms, since 50% of the genes with an intensity between 80 (4.4 ln) and 120 (4.8 ln) from MAS5 are significantly different from RMA, after normalization for signal intensity.

There were large differences between the six algorithms with respect to both gene expression and the genes that were significantly up/down regulated. This was a consequence of different ways of calculating gene expression and normalization of the microarrays. The discrepancy between the different algorithms can also be exploited. When the only aim of a microarray experiment is to select new candidate genes then the list of new candidate genes is shortened by approximately 68% by using genes predicted by all six methods. However, when the aim is to obtain expression values from a microarray experiment, a decision regarding which method to use has to be made. Various criteria were designed and tested to compare the different methods. Cope *et al*. [18] compared different methods and focused their research on the assessments of performance in terms of bias and variance. This was studied on the spike-in experiment from Affymetrix with the HGU95A chips and on a RNA dilution study by GeneLogic. These authors also wrote a program to compare other available algorithms [19]. Our study further explored the performance of individual algorithms further by five criteria: 1) comparison with spiked-in genes, 2) reproducibility, 3) biological relevance, 4) the use of MM probes, and 5) comparison with Real Time RT-PCR.

#### 1) Spike-in comparison

A commonly used RNA spike-in experiment from Affymetrix was used to test which method produced the most accurate results. In this spike-in experiment RNA of 42 genes in 14 concentrations were added to a human RNA sample. Virtually all signal intensities found in real microarray experiments are covered by the signal intensities found for the spike-in genes. In this experiment, the relation between known concentrations of spiked-in RNA and signal intensity can be compared between the six methods.

**Figure 4:**
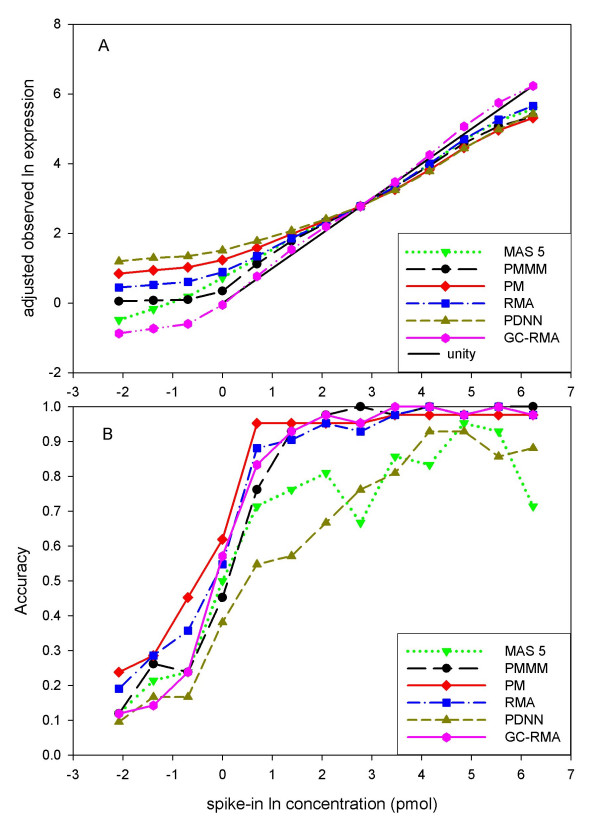
**Spiked-in data**. (**A**) Average observed ln intensity plotted against normalized ln concentration for 42 spiked-in genes of the Affymetrix spike-in experiment. The observed concentrations are adjusted so that all lines have the same intercept at a ln concentration of 2.8 (16 pmol). The solid line without symbols represents the ideal slope-1 line. (**B**) The accuracy of picking up the spiked-in genes. The significance between two successive spike-in concentrations (0–0.125; 0.125–0.25; etc.) was calculated for each gene. The number of genes where calculated per spike-in concentration that significantly where up regulated, and presented on the y-axis as percentage. This means that at "1" all 42 genes where significant at a given concentration.

The differences between the six methods were not very large between genes, which were intermediately or highly expressed (Fig 4A). Genes expressed at low levels, however, showed large differences between the six methods. PDNN and dChip PM performed worst, RMA was intermediate, and MAS5 and dChip PMMM, GC-RMA showed the most accurate results with respect to the RNA spiked-in. A good accuracy, however, should not be at the expense of precision.

As a measure of precision, the significance level was calculated between successive concentrations of all of the 42 RNA's spiked-in, so between 0 and 0.125 pM, between 0.125 and 0.25 pM up to 512 pM. Subsequently the fraction of genes that were up regulated significantly was plotted (figure 4B). For the higher concentrations of spike-in genes (>1 pmol RNA or zero on a ln scale), dChip PM, dChip PMMM, RMA and GC-RMA showed almost perfect results, this in contrast to PDNN and MAS5. The results of the ROC curves from Cope *et al*. [18] also showed that MAS5 performed worse than RMA and PM. For the genes expressed at low levels, all methods performed inadequately. Also, when all successive spike-in concentrations were compared with the zero control, again the six methods showed adequate results only at higher concentrations (>1 pmol, data not shown). This shows that although there are differences between methods in accuracy at low concentrations (Fig. 4A), these do not seem to be relevant, since no significant differences between any of the methods were observed in this lower range (Fig. 4B).

By extrapolating the results of the 42 spike-in genes and using the signal intensity from 1 pmol spike-in RNA, it is easy to calculate the number and percentage of genes which are equal to the background control (Table 3). In the normal data analysis these genes can be ignored. Depending on the method expression levels of 12 to 49% of the genes should be considered as not above background. A possible explanation for the poor results of the spike-in controls for the MAS5 and PDNN models at the higher expression levels would be the lower reproducibility of these methods. The data for the genes with the highest and lowest 10% of all the standard deviations from the spiked-in genes are excluded, then as expected MAS5 (0.131), PDNN (0.078) and GC-RMA (0.071) showed on average the highest standard deviations followed by RMA (0.055), PMMM (0.051) and PM (0.030).

#### 2) Reproducibility

**Figure 5:**
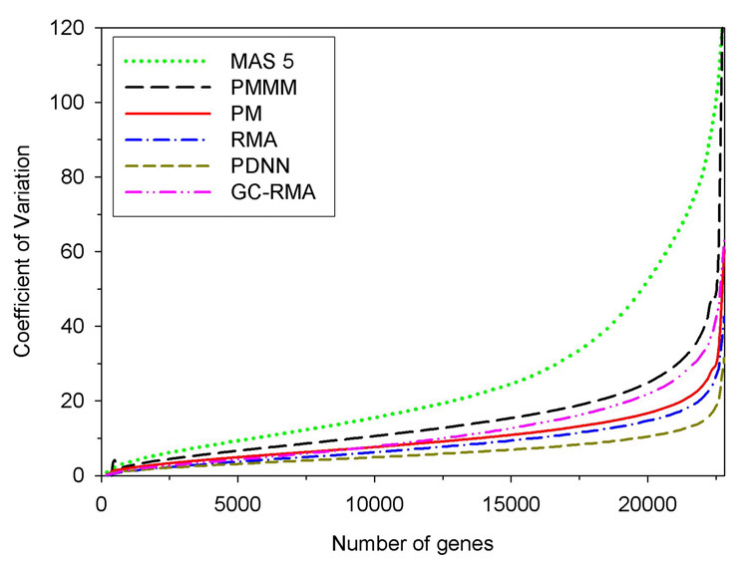
**Reproducibility**. Reproducibility of expression data between three biological replicates (air), compared between MAS 5.0, dChip PM, dChip PMMM, RMA, GC-RMA and PDNN. Reproducibility is calculated as the standard deviation divided by the average signal, which is the coefficient of variation (CV). The CV values are sorted from low to high. The PM, RMA and PDNN algorithms are giving the best reproducible results and MAS 5.0 the worst. Reproducibility of the two other replicated treatments ethylene and low-light gave similar results (data not shown).

Algorithms that are model-based can detect abnormally behaving probes more efficiently than non model-based algorithms. Therefore, gene expression values from dChip, RMA, GC-RMA and PDNN result in more reproducible results. The coefficient of variation (standard deviation/average * 100%) is a measure of reproducibility which is independent from the mean. The coefficient of variation for all genes from the three control microarrays were calculated using the six algorithms (Fig. 5). When gene expression was calculated with MAS5 algorithm, 12752 genes (56%) showed a coefficient of variation lower than 20%. Therefore, in general the results from three biological replicates were highly reproducible, with only a small portion of the genes behaving inconsistently between the replicates. As expected the model-based algorithms produced more reproducible results in that 18000 (dChip PMMM), 19209 (GC-RMA), 21167 (dChip PM), 21787 (RMA), or 22504 (PDNN) genes had a coefficient of variation lower than 20%. Furthermore, if the data for the genes with the highest and lowest 10% of all the standard deviations from all the genes were excluded, than MAS5 (0.136) showed on average the highest standard deviations, followed by PMMM (0.058), GC-RMA (0.056), PM (0.038), RMA (0.035) and PDNN (0.024). Surprisingly, the PDNN method showed the most reproducible results, which contrasted with the reproducibility of the spike-in data. Similar results were obtained with the microarray data for the ethylene or low-light treatments (data not shown). The model-based algorithms showed the best reproducibility and discarding the MM values improved the reproducibility of these methods even more. *Cope et al*. (2004) also calculated the median standard deviation for different methods and showed that RMA and PM gave better results than MAS5.

#### 3) Biological relevance

For many genes it has been unambiguously demonstrated that their expression is affected by a particular treatment or mutation. It is well known that upon addition of ethylene the expression of several genes in ethylene biosynthesis and ethylene signal transduction pathways show a consistent up-regulation. For example, the expression of ACC oxidase increases after ethylene addition in several model systems [20-22]. Also, some ethylene receptors [23-25] and the *CTR1 *gene [26] are known to be up-regulated after ethylene addition. Since the change in expression has been reported to occur within 3 hours after ethylene treatment we also expect such changes in our array data, based on a treatment of 3 hours. Data calculated using all six algorithms indeed showed up regulation of an ACC oxidase and of several ethylene receptor genes (Table 4). There were small differences between the results depending on the method used. Nevertheless, on the basis of these few genes all six methods produced biologically correct data. Therefore, this "biological relevance" criterion is not useful to differentiate between the methods until it is biologically proven how thousands of genes may respond to a given treatment.

#### 4) The usefulness of mismatch (MM) probes

MM signals on Affymetrix arrays are designed to interrogate the level of non-specific binding for the PM probes. In principle, carefully designed mismatches but not conventional probes can be used to solve the cross-hybridisation to non-target sequences from genes in large families [27]. Only Choe *et al*. [15] concluded that subtraction of the MM signal yields better results, because by subtracting the MM signals the variance at low signal-intensity levels is increased, thereby effectively reducing the false positive call. However, the use of MM values has been questioned by others [28-30,4]. Zhou and Abagyan [30] concluded that the MM values are not necessary as a previous version of MAS software, MAS 4.0, discards the one-one correspondence between a PM and its MM partner and still gives satisfactory results. A large number of MM probes show higher intensities than the corresponding PM probe and can therefore not simply be reporting background hybridisation. For this reason, Neaf *et al*. [28] concluded that MM values should not be used. According to Irizarry *et al*. [4] it is possible that information about non-specific binding is contained in the MM values, but their empirical results demonstrate that "mathematical subtraction does not translate to biological subtraction". They stated that, until a better solution is proposed, simply ignoring these values is preferable. Also, Allemeersch *et al*. [31] concluded that the MM values should be ignored because RMA outperformed MAS5 in reproducibility, dynamic range and other related criteria. In this paper we would like to discuss the use of MM values. We assessed whether identical cRNAs bind to both the PM and MM probes by i) calculating melting temperatures, and ii) analysing the relation between signals from PM and MM probes.

**Figure 6:**
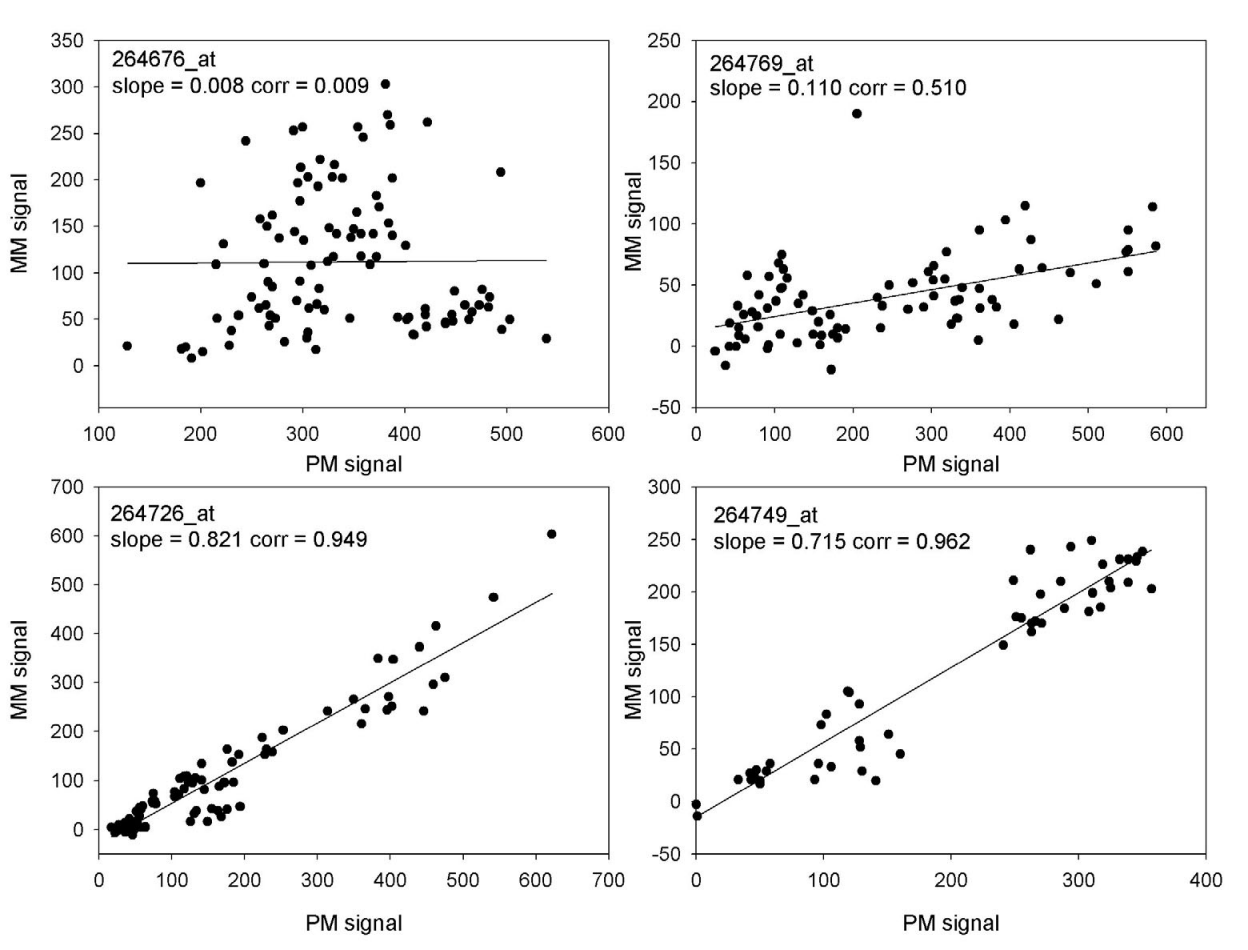
**Examples of the relation between PM and MM signals**. Relation between the PM and MM signals of four probe sets from all 9 arrays (A...D). Only the data point are plotted when the MM signal intensity is smaller than the PM signal. In panel A and B there is no correlation between the PM and MM signals as can been seen by the low slope and Pearson correlation coefficient. This in contrast to results in panel C and D were the slope and Pearson correlation coefficient are large. These signals are obtained from the microarray scanner and are the input for the six calculation methods.

i) One possible contributor to the MM values may be binding of small cRNAs to the lateral sequence of both PM or MM probes. In other words, since the difference between the PM and MM is at position 13 of an oligonucleotide 25 bases long, any cRNA that can bind to either of those lateral 12 bases might remain bound in the detection step. It is similarly likely that a cRNA fragment with its terminal 12 base pairs complementary to either lateral 12 base sequence of the probe may also remain bound at detection. In this latter case if the cRNA represents a truncated version of the expected signal cRNA then true transcript signal may be added to both PM and MM values. According to the rules regarding maximum GC content for probes described by Lockhart *et al*. [32] the experimental hybridisation for affymetrix arrays is performed at a temperature (45°C) and stringency wash (50°C) which are both higher than the maximum possible melting temperature of a 12 mer. Based on this argument, cRNAs smaller than 13 bases will not contribute to the microarray signal values. Furthermore, since the cRNAs are fragmented before hybridisation to an average length of 100 bases long the percentage of small stretches is low.

ii) One of the probe rules described by Lockart *et al*. [32] is that each probe should differ at least three bases with any other locus on the genome, to avoid cross hybridization. The MM probes are designed on purpose in such a way that they violate this rule, because MM probes differ by only one base from the PM probes. Therefore, DNA or RNA binding to the PM could possibly also bind to the MM probes and vice versa. This hypothesis is supported by the results of resequencing (or 4 L tiled) microarrays. On these arrays the position of a genomic mutation is detected by a so called "footprint", a characteristic decrease of signal for the probes flanking the mutation [33], also illustrated in figure 1 of both Chee *et al*. [33] and Wang *et al*. [34]. This loss of signal in the footprint shows clearly that a MM probe binds to the same targets as a PM probe but at a much lower efficiency or resulting signal intensity. To further test this hypothesis we checked the correlation between the MM signals and the corresponding PM signals in our data set. The MM signals of the nine arrays were plotted against the PM signals, and since each probe set has 11 probe pairs, a maximum of 99 data points were plotted per probe set. Probe pairs were only plotted when the MM signals were smaller than PM signals (Fig. 6). On average 22.4% of the probe pairs on the Arabidopsis ATH1 microarray had MM signals higher than PM signals. This is similar to results found by Naef who showed that 17% (yeast) to 35% (mouse, humans and *Drosophila*) of the MM probes have a higher signal than the PM probes. Possibly this is related to the complexity of regulatory processes like RNA editing and alternative splicing.

**Figure 7:**
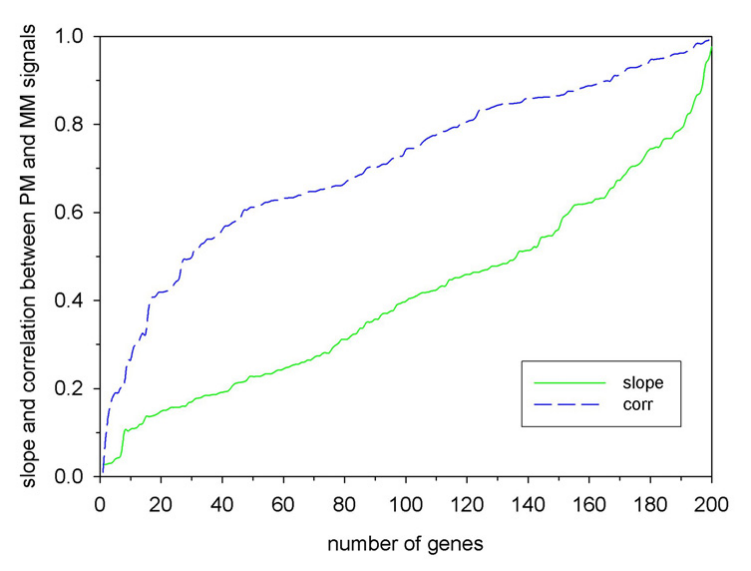
**Relation between PM and MM signals**. Slope and Pearson correlation coefficient calculated between the PM and MM signals from 200 random chosen probe sets. Only probe sets are used which represents one gene. Both slope and correlation are sorted from low to high. See figure 6 for further explanation and individual examples.

In figure 6A and 6B we show two examples in which there is clearly no correlation (Pearson correlation coefficient and slope close to 0) between the MM and PM signals and two examples (C and D) with a clear correlation (Pearson correlation coefficient and slope close to 1). It is obvious that in the latter two examples the gene expression levels of those two genes were severely underestimated. The slope and Pearson correlation coefficient of 2 × 100 randomly chosen probe sets were calculated. Only probe sets were used that represent one gene (indicated with suffix _at following the probe number), because those probe sets should have the least likelihood of cross hybridisation. The results of the first and second 100 probe sets were identical (data not shown) therefore only the result of the combined data set is shown (figure 7). From the 200 probe sets 60 had a slope larger than 0.50 and 95 probe sets had a Pearson correlation coefficient larger than 0.75. This means that in 30% of all genes the expression was underestimated with at least 50% and that in 47.5% there was a clear correlation between the MM and PM signals. Ignoring the MM probe signals lead to an increase in the calculated expression values (Table 4, Fig. 2 and 3). There was no clear relation between the level of gene expression and probe sets with a correlation between the PM and MM signals (data not shown). To our knowledge we show for the first time that many probe sets had a correlation between the MM and PM signals, and MM probes could therefore be less good estimators for the non-specific binding of PM probes. It is not clear though in which cases there really is non-specific binding, or binding of the same gene transcript to both MM and PM probes. Considering the strong correlations found, probably the same transcript is binding to both probes. If this is true then algorithms making use of MM signals can seriously underestimate the gene expression signal intensity, and as a result, different genes will be falsely identified as differentially regulated.

If MM signals are important for detecting genes expressed at low levels, differences are expected between algorithms using the MM signals (MAS, PMMM) and methods not using the MM signal. Analysis of the spike-in data shows (Fig. 4B) that MAS and PMMM are, at best, performing equally well in detecting low expressed spiked-in genes. Of the four methods that do not use the MM signals one performed poorly for highly expressed spike-in genes, compared to one out of two for the methods using the MM signals. These data suggest that the MM signals do not increase the detection of spike-in genes in this data set. Future research with independent techniques and independent data sets will have to prove how useful MM signals are.

#### 5) Real Time RT-PCR

To further test which algorithm calculates microarray gene expression most accurately, the results of the six algorithms were compared with Real Time RT-PCR on a subset of 18 genes. These 18 genes are predicted by all the six methods to be differentially regulated. Real Time RT-PCR data can also be calculated with several different algorithms. Most widely used is the ΔΔCt method [35]. One of the assumptions of this method is that the efficiencies of the PCR reactions are the same for the gene of interest and for a reference gene. More recently, other methods have become available, such as the "Assumption-free analysis" [36]. This method determines the efficiency of each PCR reaction by calculating the slope for the exponential part of the curve. Czechowski *et al*. [37] combined the two methods by using the average efficiency of a gene to correct each ΔΔCt value.

A comparison was made between three Real Time RT-PCR algorithms and six microarray algorithms. The ΔCt and the ΔCt in combination with the Assumption Free method gave highly significant correlations with all microarray methods (Table 5). The Real Time RT-PCR Assumption Free method had less significant correlations with the microarray methods. Regardless of the used Real Time RT-PCR method the best correlation was always found in combination with RMA. Moreover, the highest correlation was found for RMA [4,5] in combination with the ΔΔCt method [35]. No significant different correlations were detected. However, the used test is very conservative and will only render significant differences if the correlation coefficients are very different or if the sample sizes are much larger [[38], p582].

To test if there are differences between genes (18) with respect to what the best method for calculating expression levels, the same correlations were calculated per gene. However, due to the low number of data points per correlation (3), hardly any significant correlations were found. Therefore, we were not able to determine if there is a gene dependency for the best method. To resolve this matter, a wider range of expression levels per gene should be examined.

We also tested if the correlations between the microarray and Real Time RT-PCR data depended on signal intensity. Possibly such correlations might be weak or absent at low signal intensities. However, we did not find any significant relation between these parameters. Moreover, except for one, the signal intensities where all larger than the minimal signal intensity to detect spike in gene expression as found in table 3.

Besides comparing gene expression values from genes predicted to be changed in expression by all microarray methods we also measured Real Time RT-PCR (ΔCt) expression from 10 genes which were only shown by the RMA method to have a changed expression but not by one of the other algorithms. Of these 10 genes, 5 showed the same change in direction (either up- or down regulated) with Real Time RT-PCR upon ethylene treatment (table 6). From those 5 genes, only one showed a significant change in expression. There could be two reasons why the RMA microarray data poorly corresponds with the Real Time RT-PCR data. First, the changes in expression of genes exclusively detected with RMA are rather small (on average -28 or +48%). Differences of this magnitude are difficult to detect with Real Time RT-PCR. Second, the standard deviation of the calculated gene expression is compressed by RMA, which could result in a high number of false positives. So while the correlations suggest that RMA is the best method, for the most part we could not verify expression changes exclusively detected by RMA with Real Time RT-PCR.

## Conclusion

Gene expression was calculated with six algorithms, MAS5, dChip PMMM, dChip PM, RMA, GC-RMA and PDNN. All six algorithms resulted in different levels of gene expression, with MAS5 calculating the lowest and PDNN the highest signals. Consequently, depending on the method used, different genes will be identified as up or down regulated by a given treatment. Therefore, it is up to the user to decide which method to use to calculate the gene expression profile. The spike-in experiment showed that the differences between the methods for the low expressed genes are not relevant since measurements up to 1 pmol RNA, did not result in significant signals above background. Furthermore, for relatively high expressed genes MAS5 and PDNN showed the lowest percentage of correctly calculated genes. In our comparison all six methods yielded the expected biological results, with respect to ethylene-induced gene expression. Furthermore, all model-based algorithms produced more reproducible results in comparison to MAS5. Moreover, we conclude that MM signals do not represent non-specific binding for PM signals, since in 47.5% of the cases there was a significant correlation between the MM and PM signals. Subtracting the MM from the PM signals will therefore most likely underestimate the expression signal. RMA generally yields the most reproducible results. However, we were not able to verify most genes which were solely identified as having changed expression by RMA with Real Time RT-PCR. Different experimental systems may require different methods to obtain the best result [16]. If the only purpose of the microarray experiment is to find new candidate genes, and too many genes are found, then mutual exclusion of the genes predicted by contrasting methods can be used to narrow down that list of candidate genes.

## Methods

### Plant material and growth conditions

*Arabidopsis thaliana *accession Columbia-0 (Col-0; N1092, Nottingham Arabidopsis Stock Centre) was used. Seeds were sown on moistened filter paper in sealed Petri-dishes and cold-stratified in the dark at 4°C for 4 d. Subsequently, the seeds were germinated for 4 d in a growth chamber with the following conditions: 20°C, 70% (v/v) relative humidity, 9 h photoperiod: 200 μmol m^-2 ^s^-1 ^photosynthetic active radiation (PAR). Seedlings were transferred with a brush to pots (70 ml) containing a mixture of potting soil and perlite (1:2, v:v), enriched with 0.14 mg MgOCaO (17%; Vitasol BV, Stolwijk, The Netherlands) and 0.14 mg slow-release fertilizer (Osmocote "plus mini"; Scotts Europe bv, Heerlen, The Netherlands) per pot. Prior to seedling transfer, to each pot 20 ml nutrient solution was added, containing: 2.6 mM KNO_3_, 2.0 mM Ca [NO3]_2_, 0.6 mM KH_2_PO_4_, 0.9 mM MgSO_4_, 6.6 μM MnSO_4_, 2.8 μM ZnSO_4_, 0.5 μM CuSO_4_, 66 μM H_3_BO_3_, 0.8 μM Na_2_MoO_4 _and 134 μM Fe-EDTA, pH 5.8. All chemicals were p.a. grade and obtained from Merck (Darmstadt, Germany). Following transplantation, plants were grown for 28 d in a growth chamber (conditions as described above). Pots with seedlings were kept in a glass-covered tray for the first 4 d following transplantation, after which they were transferred to irrigation mats (Maasmond-Westland, The Netherlands). The mats were automatically watered with tap water to saturation once a day (at the beginning of the light period), and the excess water was drained.

### Ethylene and low-light experiments

Ethylene (100 μL L^-1^; Hoek Loos BV, The Netherlands) and air (70% relative humidity) were mixed using flow meters (Brooks Instruments BV, The Netherlands) to generate a concentration of 5 μL L^-1 ^ethylene, which was flushed continuously through glass cuvettes (13.5 × 16.0 × 29.0 cm) at 75 L h^-1^, and then vented to the outside of the building. This concentration saturates ethylene-induced petiole elongation in *R. palustris *[39]. A concentration of 1 μL L^-1 ^ethylene was reached in the cuvettes after approximately 10 min of starting the treatment; 5 μL L^-1 ^was reached after 40 min. The ethylene concentration was checked regularly on a gas chromatograph (GC955, Synspec, Groningen, Netherlands), and remained constant for the duration of the experiment. Control cuvettes were flushed with air (70% relative humidity) at the same flow rate.

For the shading treatment, the light quantity was reduced by 90% to 15 – 20 μmol m^-2 ^s^-1 ^(photosynthetic active radiation) at the start of the experiment. This was achieved by switching off a number of lamps in the growth chamber and by using a mesh black spectrally neutral shade cloth. These alterations did not change the light quality when checked with a Licor1800 spectro-radiometer (Li-cor, Lincoln, Ne, USA) (data not shown).

### RNA isolation

Petioles were harvested and directly frozen in liquid nitrogen from plants in stage 3.9 according to Boyes *et al*. [40], which means a plant with approximate 17 rosette leaves. Subsequently RNA was isolated from these petioles with RNeasy extraction protocol from Qiagen (Valencia, CA, USA).

### cDNA synthesis

Approximately 10 μg of total RNA was reverse transcribed at 42°C for 1 h to generate first strand DNA using 100 pmol oligo-dT(24) primer containing a 5'-T7 RNA polymerase promoter sequence (5'-GGCCAGTGAATTGTAATACGACTCACTATAGGGAGGCGG-(dT)24-3'), 50 mM Tris-HCl (pH 8.3), 75 mM KCl, 3 mM MgCl_2_, 10 mM dithiothreitol (DTT), 10 mM dNTPs and 200 units SuperScript II reverse transcriptase (Invitrogen Life Technologies, Breda, The Netherlands). Following first strand synthesis, second strand DNA was synthesized using 10 units of *E. coli *polymerase I, 10 units of *E. coli *DNA ligase and 2 units of RNase H in a reaction containing 25 mM Tris-HCl (pH 7.5), 100 mM KCl, 5 mM MgCl_2_, 10 mM (NH_4_)SO_4_, 0.15 mM b-NAD+ and 10 mM dNTPs. The second strand synthesis reaction proceeded at 16°C for 2 hours before 10 units of T4 DNA polymerase was added and the reaction allowed to proceeded for 5 minutes. The reaction was terminated by adding 0.5 M EDTA. Double stranded cDNA products were purified using the GeneChip Sample Cleanup Module (Affymetrix, USA).

### cRNA synthesis

The synthesized cDNAs were *in vitro *transcribed by T7 RNA polymerase (ENZO BioArray High Yield RNA Transcript Labeling Kit, Enzo, San Francisco, CA, USA) using biotinylated nucleotides to generated complementary RNAs (cRNAs). The cRNAs were purified using the GeneChip Sample Cleanup Module (Affymetrix, USA). The cRNAs were then randomly fragmented at 94°C for 35 min in a buffer containing 40 mM Tris-acetate (pH 8.1), 100 mM potassium acetate, and 30 mM magnesium acetate to generate molecules of approximately 35 to 200 bases long.

### Array hybridization

The fragmented cRNA were mixed with 0.1 mg ml^-1 ^of sonicated herring sperm DNA in a hybridisation buffer containing 100 mM 2-N-morpholino-ethane-sulfonic acid (MES), 1 M NaCl, 20 mM EDTA and 10% Tween-20 making a hybridisation mixture. The hybridisation mixture containing the fragmented cRNA was denatured at 99°C for 5 min and equilibrated at 45°C for 5 min and centrifuged at maximum speed in a microcentrifuge for 5 min to remove any insoluble material from the hybridisation mixture. The hybridisation mix was transferred to the Arabidopsis ATH1-121501 genome array (Affymetrix) cartridge and hybridised at 45°C for 16 h on a rotisserie at 60 rpm. After a 16 h hybridisation period, the arrays were washed and stained in a fluidics station (Affymetrix). The arrays were initially washed in a low stringency buffer A (6× SSPE (0.9 m NaCl, 0.06 M NaH_2_PO_4_, 0.006 M EDTA), 10% Tween 20) at 25°C for 10 min and then incubated with a high stringency buffer B (100 mM MES, 0.1 NaCl, 10% Tween-20) at 50°C for 20 min, then stained with 10 mg ml^-1 ^of Streptavidin Phycoerythrin (SAPE), in stain buffer containing 100 mM MES, 1 M NaCl, 0.05% Tween 20 and 2 mg ml^-1 ^BSA at 25°C for 10 min, washed with wash buffer A at 25°C for 20 min and stained with biotinylated anti-streptavidin antibody at 25°C for 10 min. After antibody staining the arrays were stained again with SAPE for signal amplification and washed with buffer A at 30°C for 30 min. The arrays were finally scanned and the intensities averaged with a Agilent GeneArray Scanner.

### Real time RT-PCR

For one RNA sample eight petioles obtained from two plants were mixed and ground in liquid nitrogen. Samples were taken after 3 h of ethylene treatment or from plants in air for 3 h. Total RNA was isolated from *Arabidopsis thaliana *petioles using RNeasy Plant Mini Kit (Valencia, CA, USA). Genomic DNA was removed using the DNA-Free kit (Ambion, Cambridgeshire, United Kingdom). cDNA was synthesized using 3.3 μg total RNA with Superscript III RNase H^- ^Reverse Transcriptase (Invitrogen, Breda, The Netherlands) using Random-Hexamer Primers. Real time PCR reactions were performed on MyiQ Single-Color Real-Time PCR Detection System and Software using iQ SYBR Green Supermix fluorescein (Bio-Rad laboratories, Veenendaal, The Netherlands).

Real time PCR was conducted (12.5 μL SYBR Green Supermix fluorescein, 1 μL from each primer (100 pmol), 1 μL cDNA, 9.5 μL water) for 40 cycles with the following temperatures: 30 sec 95°C denaturation, 30 sec 60°C annealing, and 60 sec 72°C extension. Melt curves showed single products for all samples (data not shown). All mRNA expression values are relative to Actin 2 (At5g09810) that is constitutively expressed in vegetative structures, and is not influenced upon the treatment (data not shown). See table 7 for the primers used, the length of the PCR products and the gene codes.

### Spike-in experiment

The RNA spike-in experiment is carried out by Affymetrix on Human HG-U133 chips. The CEL files are available online [17]. In this experiment the RNA of 42 genes are spiked-in at 14 concentrations 0, 0.125, 0.25, 0.5, 1, 2, 4, 8, 16, 32, 64, 128, 256, 512 pmol and in total 42 micro arrays were used. For more details see the Affymetrix website.

### Data analysis

The following programs are used, MAS 5.0 from Affymetrix (Santa Clara, USA), DNA-Chip analyser (dChip) version 1.2 [41], RMA version 0.2 [42], PDNN version 2.3 [43], GC-RMA as was incorporated in the program ArrayAssist Lite version 3.2.1954.31591 (Stratagene, La Jolla, CA, USA) and Excel 2000 (Microsoft, Redmond, USA). Cel files from MAS5 containing the signal intensity per feature were used as input for dChip, RMA and PDNN. A two-sided T-test with unequal variances was performed in Excel with a p value of 0.05 unless stated otherwise. The coefficient of variation or CV is calculated in Excel as the standard deviation divided by the average signal. The CV was sorted separately for each algorithm before the distribution of the CV was plotted; this was also the procedure for the signal distributions, slope and correlation data in figure 2, 5, and 7. A macro was written in Excel to find common genes between two sets of genes to construct figure 1, this macro is available [44].

Real Time RT-PCR data were calculated in three ways: 1) comparative Ct method described by Livak and Schmittgen [35], 2) "Assumption-free analysis" by Rademakers *et al*. [36] and 3) a combination of the first two methods by Czechowski *et al*. [37].

## Authors' contributions

FFM performed the analyses and wrote the manuscript. JO and STM were responsible for the microarray hybridization, and for constructive discussions. MvZ preformed the Real Time RT-PCR. LACJV and AJMP have been involved in revising this manuscript, and for constructive discussions. All authors read and approved the final manuscript.

## Figures and Tables

**Table 1:** The number of differentially expressed genes. Number of genes significant (Ttest p < 0.05) up or down regulated between air control and ethylene or low-light treatment in six different algorithms. The number of biological replicates for each treatment is three. The number of probe pairs per chip is 22746.

Method	Control – ethylene	Control – Low-light
MAS 5.0	2201	2384
dChip PMMM	2559	2665
dChip PM	2435	2583
RMA	2470	2705
GC-RMA	2258	2411
PDNN	2972	3262

**Table 2:** Correlations between the expression values. Pearson correlation between the expression values calculated with six different methods. The average signal intensity from all 22746 probe sets from the three control chips was used.

	MAS5	dCHip PMMM	dCHip PM	RMA	GC-RMA
dCHip PMMM	0.9831				
dCHip PM	0.9846	0.9889			
RMA	0.9913	0.9778	0.9862		
GC-RMA	0.9435	0.9325	0.9391	0.9417	
PDNN	0.9767	0.9736	0.9793	0.9764	0.9534

**Table 3:** Minimum signal. Signal intensity from the RNA spike-in experiments at a concentration (1 pmol) where the spiked-in concentrations were significant different from the background. The number (#) and percentage (%) genes below the minimal signal (Min. signal) is obtained from non-transformed data.

	MAS5	dCHip PMMM	dCHip PM	RMA	GC-RMA	PDNN
Min signal	40.4	46.5	112.2	59.2	27.4	261.3
# genes	11109	2651	3895	6750	9174	2651
% genes	48.8	11.7	17.1	29.7	40.3	11.7

**Table 4:** Biological comparisons. Expression of genes in ethylene biosynthesis and signal transduction cascade as calculated with six different methods. Numbers in brackets after a gene name show how many genes of a family are on the microarray, followed by the size of the family. Expression data of a gene family is the summation of expression of the individual gene members. The columns with a p showing the significance of the fold change ('x' columns) between air control and ethylene (eth) treated plants. ns = not significant * = p < 0.05, ** = p < 0.01, *** = p < 0.001.

	MAS 5.0		dChip PMMM				dChip PM					
	
gene	Air	eth	p	x	Air	eth	p	X	Air	eth	p	x
ACC synthase (10/11)	378	339	ns	-	1882	1760	*	0.9	2489	2373	ns	-
ACC oxidase (10/10)	2960	5845	**	2.0	6196	12866	**	2.1	8301	16931	***	2.0
Receptors (5/5)	327	932	**	2.9	806	2104	**	2.6	1331	2881	***	2.2
*CTR1*	55	242	***	4.4	120	614	***	5.1	278	877	***	3.1
*EIN2*	191	182	ns	-	429	415	ns	-	588	558	ns	-
*EIN3/EIL *(4/4)	578	652	**	1.1	1464	1714	**	1.2	2075	2377	**	1.1
												
	RMA		PDNN				GC-RMA					
	
gene	Air	eth	p	x	Air	eth	p	X	Air	eth	p	x
ACC synthase (10/11)	1304	1186	ns	-	4337	4057	*	0.9	801	567	***	0.7
ACC oxidase (10/10)	7341	15768	***	2.2	14731	28773	***	2.0	9116	23075	***	2.5
Receptors (5/5)	869	2150	***	2.5	2419	5059	***	2.1	600	2221	***	3.7
*CTR1*	210	904	***	4.3	558	1566	***	2.8	140	1031	***	7.4
*EIN2*	608	563	ns	-	1029	974	ns		407	369	ns	0.9
*EIN3/EIL *(4/4)	1493	1740	***	1.2	3678	4416	*	1.2	1785	2207	***	1.2

**Table 5:** Correlation between microarray and RT-PCR data. Pearson correlation of expression values between six microarray algorithms and three Real Time RT-PCR algorithms on a subset of 18 genes. The negative sign from the numbers in the second and last column are removed, because a lower ΔCt value means more gene product present, consequently leading to a negative correlation with microarray data. All correlations were significant. ns = not significant (p < 0.01). * = p < 0.05, ** = p < 0.01

	ΔCt	Assumption Free	ΔCt + Assumption Free
MAS 5.0	0.515**	0.311*	0.406**
dChip PMMM	0.468**	0.298*	0.382**
dChip PM	0.460**	0.300*	0.397**
RMA	0.554**	0.378**	0.459**
GC-RMA	0.495**	0.297*	0.407**
PDNN	0.462**	0.252^ns^	0.386**

**Table 6:** RT-PCR data of RMA specific genes. Ethylene/air expression signals from 10 genes calculated with RMA and compared to the Real Time RT-PCR data as calculated with ΔCt. All RMA signals from ethylene treated plants are significantly (0.05 > p > 0.01) different from the air controls (average p = 0.037). The Real Time RT-PCR data shown, are averages, standard deviations and p values after comparison with the control. The average control signal is set to 1. The numbers in bold indicate genes which show a change in expression similar for both methods. For RMA, n = 3 and for Real Time RT-PCR, n = 3–5.

AGI	Eth/air RMA	Eth/air RT-PCR		
	average	average	stdev	p
At5g23060	**0.65**	**0.83**	0.075	0.456
At2g36850	**0.72**	**0.76**	0.135	0.181
At1g70710	**0.73**	**0.82**	0.089	0.155
At5g03760	**0.78**	**0.54**	0.109	0.021
At3g49530	1.29	0.99	0.134	0.958
At2g31800	1.31	0.72	0.104	0.065
At5g25930	**1.35**	**1.22**	0.523	0.700
At1g77380	1.44	0.84	0.066	0.270
At5g57120	1.51	0.72	0.227	0.356
At1g64710	1.99	0.96	0.132	0.804

**Table 7:** RT-PCR primers. Primers for the genes used in Real-Time RT-PCR and the length of the PCR product. Each gene is represented with the Affymetrix probe number and the Arabidopsis Genome Initiative (AGI) code.

probe	AGI	length (bp)	Forward primer	Reverse primer
Genes common for all microarray methods				

245098_at	At2g40940	148	5'-CGGAACTCAGAGGAACCATT-3'	5'-GCAGATACCAAGCCTGATGA-3'
245264_at	At4g17245	50	5'-CAAGACGGTGACACCTTACG-3'	5'-TGGAATCCATGTTTGCATCT-3'
247486_at	At5g62140	158	5'-ACGGAGGAATCGATAGGAGA-3'	5'-TGTAGATCGGCGAGACACTC-3'
247954_at	At5g56870	96	5'-CAGAGAGTTCCGGTGTGAGA-3'	5'-TTCCTGCTGGTGTAGCAAAC-3'
249125_at	At5g43450	99	5'-GTTCTTGAGCGTGGAGCATA-3'	5'-ACCGTGGAATTTGGAGAAAG-3'
250598_at	At5g07690	75	5'-AACAAGCGTTGATGAAGACG-3'	5'-AAATCGGAATGGTCAAGGAG-3'
250911_at	At5g03730	67	5'-CGAGATGAGCCGTCTAATGA-3'	5'-TAGCAAGCTCCCACAAGATG-3'
251058_at	At5g01790	133	5'-AGAAACTGTCGGCTTCATCA-3'	5'-TCAGCAGAAGAGTCGAAGGA-3'
251373_at	At3g60530	129	5'-ACCGCTTGGACCTAAAACAC-3'	5'-TTCCGATGAGAGTTCGAATG-3'
253302_at	At4g33660	83	5'-ACCACCACGAAAAGTTGGTT-3'	5'-GGTCACAGCAACATTCATCC-3'
254371_at	At4g21760	51	5'-TTCATCTTCCAGCACAGAGC-3'	5'-TTGTTTCAGGCACCAATCAT-3'
258181_at	At3g21670	96	5'-GCTTACGTTGGACAGCTTGA-3'	5'-TCCCATCGATATCGTGCTTA-3'
258468_at	At3g06070	100	5'-GTTCCTTTATCCCCAAGCAA-3'	5'-GCATGATGAAAGGTGATGCT-3'
259982_at	At1g76410	142	5'-TGGCTTGGATCACACTCTTC-3'	5'-CAGGACCATCTTCACGTTGT-3'
263653_at	At1g04310	83	5'-ACGCTTGCCAAAACATTGTA-3'	5'-TGAGACGCTTTTCACCAAAC-3'
264624_at	At1g08930	135	5'-TGGAATGCATCAGGAATGTT-3'	5'-TTGCACAGAGTTGTTGAGCA-3'
265194_at	At1g05010	59	5'-TATAATCCGGGAAGCGACTC-3'	5'-GCTTCTTTTCCGATCAGCTC-3'
266884_at	At2g44790	152	5'-ACTCCTACCACACCGGAATC-3'	5'-ATCGAGACTCCCACCAAAAC-3'
RMA specific genes				
262870_at	At1g64710	129	5'-CCGATGGAAAGACCAGATTC-3'	5'-GAAACAGAGGGTCCACCTTG-3'
260181_at	At1g70710	107	5'-ACACAAAGCCTCGAGGAAAC-3'	5'-CATGCTTATTGTCCCAACCA-3'
246389_at	At1g77380	61	5'-AATGTACATCGCGCAGAAGA-3'	5'-GACTTGAAGGCAAACCCATC-3'
263461_at	At2g31800	52	5'-GGGACCTTGGGAGCTATCTT-3'	5'-ACTTTGGCTGGAGAAAGACG-3'
263891_at	At2g36850	139	5'-ATGGCTACTCGTGGGTTGTT-3'	5'-CCAAGGACACATGCAAACAT-3'
267592_at	At2g39710	128	5'-CGTATTGCATATCCGGTTCA-3'	5'-CCGGTCGAAATAAGGTAACG-3'
252278_at	At3g49530	73	5'-CGTGACCGGTTTTGTGTTTA-3'	5'-TACTGCCGCCCTAAAGAGTC-3'
250892_at	At5g03760	77	5'-CTGCTTGTGGACTCTCATGG-3'	5'-TTTGATCGTTGGATCAGTGG-3'
249876_at	At5g23060	103	5'-GCACAACAGACGTCAAAAGC-3'	5'-CAGCAGCAACAACAATGACA-3'
249822_at	At5g23710	52	5'-CGGGAAAAAGTTCGGTAATG-3'	5'-AATCGAAGCAAAAGCTCCAG-3'
